# Supplementary material for: Enhanced Efinaconazole Permeation and Activity Against Trichophyton rubrum and Trichophyton mentagrophytes with a Self-Nanoemulsifying Drug Delivery System
Source: Pharmaceutics. 2025 Sep 22;17(9):1230. doi: 10.3390/pharmaceutics17091230 (PMC12473344; doi:10.3390/pharmaceutics17091230)
Supplement: Supplementary file 1 [file pharmaceutics-17-01230-s001.zip › pharmaceutics-3871174-supplementary.pdf]

Supplementary Information

Enhanced Efinaconazole Permeation and Activity against *Trichophyton rubrum* and *Trichophyton mentagrophytes* with a Self-Nanoemulsifying Drug Delivery System

Seo Wan Yun, Jeong Gyun Lee, Chul Ho Kim * and Kyeong Soo Kim *

Department of Pharmaceutical Engineering, Gyeongsang National University, 33 Dongjin-ro, Jinju 52725, Republic of Korea; wyjm2013@naver.com (S.W.Y.); leepipi87@naver.com (J.G.L.)
* Correspondence: chkim63@gnu.ac.kr (C.H.K.); soyoyu79@gnu.ac.kr (K.S.K.)

***Co-corresponding author:**

Prof. Dr. Chul Ho Kim

Department of Pharmaceutical Engineering, Gyeongsang National University, 33 Dongjin-ro, Jinju 52725, Republic of Korea, Tel: +82-55-772-3395, Fax: +82-55-772-3399, E-mail: chkim63@gnu.ac.kr

****Corresponding author:**

Prof. Dr. Kyeong Soo Kim

Department of Pharmaceutical Engineering, Gyeongsang National University, 33 Dongjin-ro, Jinju 52725, Republic of Korea, Tel: +82-55-772-3391, Fax: +82-55-772-3399, E-mail: soyoyu79@gnu.ac.kr

Table S1. Data for EFN particle size and PDI values in water dispersions of SNEDDS compositions.

Formulation no.	Vehicle composition (Weight ratio %)			Particle size (nm)	PDI
	Kollisolv MCT 70	Solutol HS15	Labrafil M2125 CS		
F1	30	60	10	44.21 ± 0.48	0.24 ± 0.01
F2	30	65	5	37.38 ± 0.40	0.17 ± 0.02
F3	25	60	15	52.33 ± 0.96	0.25 ± 0.01
F4	25	65	10	51.32 ± 19.64	0.19 ± 0.06
F5	25	70	5	51.31 ± 23.82	0.20 ± 0.07
F6	20	60	20	36.39 ± 0.20	0.14 ± 0.01
F7	20	65	15	34.41 ± 1.18	0.24 ± 0.03
F8	20	70	10	41.68 ± 0.36	0.31 ± 0.02
F9	20	75	5	45.48 ± 23.61	0.25 ± 0.13
F10	15	60	25	40.93 ± 10.02	0.17 ± 0.07
F11	15	65	20	36.45 ± 8.56	0.20 ± 0.09
F12	15	70	15	98.29 ± 10.91	0.20 ± 0.10
F13	15	75	10	74.61 ± 20.98	0.34 ± 0.07
F14	15	80	5	42.00 ± 13.64	0.25 ± 0.09
F15	10	60	30	33.41 ± 0.76	0.18 ± 0.02
F16	10	65	25	29.06 ± 0.17	0.21 ± 0.07
F17	10	70	20	34.01 ± 1.13	0.22 ± 0.02
F18	10	75	15	25.43 ± 0.80	0.20 ± 0.01
F19	10	80	10	23.97 ± 2.75	0.18 ± 0.08
F20	10	85	5	29.23 ± 0.24	0.28 ± 0.02
F21	5	60	35	37.52 ± 0.27	0.19 ± 0.01
F22	5	65	30	34.10 ± 3.29	0.20 ± 0.01
F23	5	70	25	34.50 ± 1.60	0.23 ± 0.01
F24	5	75	20	31.04 ± 9.56	0.13 ± 0.02
F25	5	80	15	33.78 ± 0.60	0.30 ± 0.01
F26	5	85	10	36.29 ± 0.59	0.38 ± 0.01
F27	5	90	5	36.99 ± 7.64	0.38 ± 0.19

Table S2. Penetrant rating chart for permeability coefficient (K_p) based on Marzulli's definition.

Permeability constant (K_p)	Penetrant rating
$K_p > 6 \times 10^{-3}$	Very fast
$6 \times 10^{-4} < K_p \leq 6 \times 10^{-3}$	Fast
$6 \times 10^{-5} < K_p \leq 6 \times 10^{-4}$	Moderate
$6 \times 10^{-6} < K_p \leq 6 \times 10^{-5}$	Slow
$K_p \leq 6 \times 10^{-6}$	Very slow

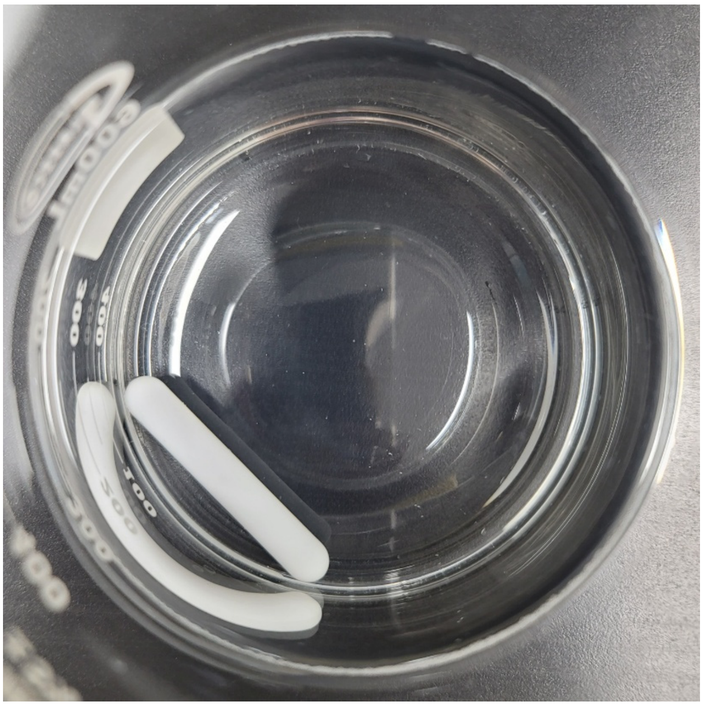

Figure S1. Representative photograph of the optimized EFN-loaded SNEDDS (F24, Kollisolv MCT 70:Solutol HS 15:Labrafil M2125 CS = 5:75:20, *w*/*w*/*w*) showing a clear nanoemulsion upon dilution.
